# Curcumin, Gut Microbiota, and Neuroprotection

**DOI:** 10.3390/nu11102426

**Published:** 2019-10-11

**Authors:** Francesco Di Meo, Sabrina Margarucci, Umberto Galderisi, Stefania Crispi, Gianfranco Peluso

**Affiliations:** 1 Institute of Biosciences and BioResources-UOS Naples CNR, Via P. Castellino, 80100 Naples, Italy; francesco.dimeo.90@gmail.com; 2 Department of Biology, University of Naples Federico II, Complesso Universitario Monte Sant’Angelo via Cinthia, 80100 Naples, Italy; 3 Institute of Research on Terrestrial Ecosystems, 05010 Porano TR, Italy; sabrina.margarucci@cnr.it; 4 Department of Experimental Medicine, University of Campania “Luigi Vanvitelli”, Via Santa Maria di Costantinopoli, 80100 Naples, Italy; umberto.galderisi@unicampania.it

**Keywords:** curcumin, gut microbioma, polyphenols, bioactivity, metabolism, neuroprotection

## Abstract

Curcumin, a nontoxic, naturally occurring polyphenol, has been recently proposed for the management of neurodegenerative and neurological diseases. However, a discrepancy exists between the well-documented pharmacological activities that curcumin seems to possess in vivo and its poor aqueous solubility, bioavailability, and pharmacokinetic profiles that should limit any therapeutic effect. Thus, it is possible that curcumin could exert direct regulative effects primarily in the gastrointestinal tract, where high concentrations of curcumin are present after oral administration. Indeed, a new working hypothesis that could explain the neuroprotective role of curcumin despite its limited availability is that curcumin acts indirectly on the central nervous system by influencing the “microbiota–gut–brain axis”, a complex bidirectional system in which the microbiome and its composition represent a factor which preserves and determines brain “health”. Interestingly, curcumin and its metabolites might provide benefit by restoring dysbiosis of gut microbiome. Conversely, curcumin is subject to bacterial enzymatic modifications, forming pharmacologically more active metabolites than curcumin. These mutual interactions allow to keep proper individual physiologic functions and play a key role in neuroprotection.

## 1. Introduction

Over the last decades, the interest in herbal medicines has greatly increased since they can exert preventive effects against chronic and degenerative diseases including cancer. In addition, these molecules play an important role in neuroprotection by modulating different cellular functions. Different studies have shown that curcumin, like others dietary polyphenols, is able to counteract the effects of toxic damage in different tissues [1,2,3].

Polyphenols are a large class of compounds (curcumin, lignans, lignins, stilbenes, flavonoids, coumarins, cinnamic acid, benzoic acid, etc.) which possess at least one aromatic ring structure with one or more hydroxyl groups. They are present in most vegetables and secondary metabolites and derive from the shikimic acid pathway. Contrasting findings on the bioavailability of polyphenols created doubts about their usefulness as beneficial antioxidant compounds. Currently, there are findings showing that polyphenols can exert their biological effects following chemical modifications performed by gut microbiota. Indeed, enzymes of the gut microbiota can perform deglycosylation, dihydroxylation, and ademethylation of polyphenols that result in small catabolic products, which may be easily absorbed during intestinal transit. These catabolites may fall in two classes: some have higher biological activities compared with “parental” compound, and others lose biological activity [4].

Among polyphenols, curcumin has received great attention by researchers in the last years. Curcumin is normally found in the turmeric of *Curcuma longa* Linn. This plant belongs to the Zingiberaceae family and it is native from South Asia. Turmeric has been used in Asia from ancient times in traditional medicine and nowadays it is widely used in food, cosmetic, and pharmaceutical industries [5].

Turmeric contains essential oils such as zingiberene [6] and coloring agents that are known as curcuminoids [7]. Curcumin is a polyphenol that represents the most important curcuminoid isolated from the rhizome of the plants. It has been used for centuries as a herbal remedy for the treatment of several diseases in East Asia [8,9,10] due to its safety and intrinsic nontoxicity to humans, even at high doses [11].

The general interest in its therapeutic efficacy arises from the different biological and pharmacological effects. Curcumin possesses antioxidant [12,13] and anti-inflammatory properties [14,15], and it has shown to exert beneficial effects against several types of cancers [16,17,18].

It should also be highlighted that an increasing number of clinical trials based on curcumin administration have been published or are currently in progress, therefore demonstrating the expanding interest of the scientific community on the therapeutic potential of curcumin [8,19].

However, the pharmacological potential of curcumin is widely restricted because of its poor water solubility, chemical instability, and rapid metabolism. In addition, curcumin bioavailability is very low after oral administration [20,21]. In this regard, numerous attempts have been made to increase its efficacy and bioavailability. To overcome solubility problems, different curcumin formulations using nanocarriers have been tested [22,23,24]. Other strategies developed to increase curcumin bioavailability are based on combined administration of curcumin with other molecules. For example, the co-administration of curcumin with piperine, an alkaloid of black pepper and long pepper, significantly enhances curcumin bioavailability since piperine prevents curcumin metabolism through the inhibition of the glucuronidation processes [25,26].

The puzzling between the poor bioavailability and the large variety of pharmacological activities of curcumin can be solved by taking into account the reciprocal interactions between curcumin and the gut microbiota.

Gut microbiota is one of the most densely and dynamic populated microbial ecosystems that contribute to individual health. Gut microbiota composition changes with age and it is strictly related to diet. Different unbalanced diets determine alterations in the gut microbiota composition, resulting in modification of gut permeability and in gut low-grade inflammation [27].

Like other dietary polyphenols, curcumin bioactivity is related not only to the absorption rate, but also to its metabolism that is due to the digestion from intestinal microbes. Biological activity of the derivative metabolites may differ from the native curcumin. In addition, specific biological properties often depend on bioactive metabolites produced by gut microbiota digestion [28]. In this regard, it is worthy to note that curcumin, after administration, accumulates in the gut where, after microbial digestion, it can be transformed into biologically active metabolites [29,30,31].

In this review, we discuss the curcumin–gut microbiota interplay that allows transforming dietary curcumin into bioactive derivatives and how these molecules can exert neuroprotective functions.

## 2. Curcumin Metabolism

The bioavailability of curcumin, as with other polyphenols, is generally poor and after oral dosing, its blood levels are extremely low.

The oral bioavailability of curcumin is low due to a relatively low intestinal absorption and the rapid metabolism in the liver, followed by elimination through the gall bladder. An oral dose of 0.1 g/kg administered to mice yielded a peak plasma concentration of free curcumin that was only 2.25 μg/mL [32]. In a clinical trial with an oral dose of 3.6 g of curcumin, a plasma level as low as 11.1 nmol L^−1^ was detected an hour after oral dosing [33]. Also, after ingestion of high doses of curcumin, 8 g per day via oral administration, the plasmatic level of free curcumin was negligible [34].

Curcumin is used in different dosage depending on the disease. Recent in vitro studies report that curcumin is effective in reducing oxidative stress (OS) and in preventing neurodegeneration when used at a concentration ranging from 5–50 μM [35,36] and at a dose from 50 to 200 mg/Kg/day in vivo [37,38,39]. In clinical trials, curcumin is effective on oxidative stress and inflammation at a dosage from 90 to 2000 mg/day [40,41,42], while in neurodegenerative diseases, prevention is evident at a dosage of 500–2000 mg [43].

**Figure 1:**
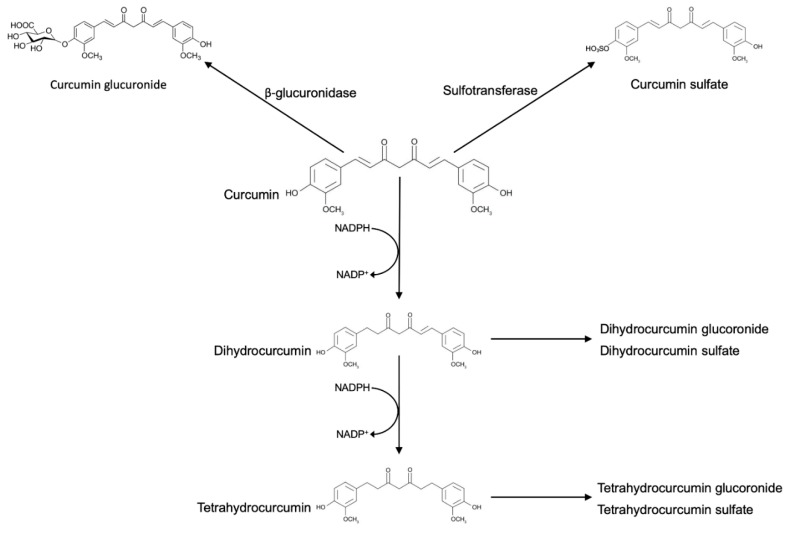
Reductive and conjugative metabolism of curcumin. Glucuronidation and sulfation are the predominating pathways of conjugation. Curcumin is also reduced by endogenous reductase systems in a stepwise manner and subsequently, curcumin metabolites are glucuronidated and sulfurated.

Turmeric contains about 2%–9% of curcuminoids. Commercial turmeric extracts contain approximately 70%–75% curcumin, 20% demethoxycurcumin, and 5% bisdemethoxycurcumin. Chemically, curcumin is a β-diketone α- β-unsaturated ferulic acid. Curcumin shows a keto–enol tautomerism, and the balance between the two forms depends on the polarity and pH of the solvent with the keto and enol forms existing in given proportions. Once dissolved, the enol form predominates (Figure 1). Curcumin is soluble in ethanol, acetic acid, dichloromethane, chloroform, methanol, ethyl acetate, dimethyl sulfoxide, and acetone, while it is insoluble in water [44].

The bioavailability of curcumin has been studied extensively in mouse and humans. After oral administration of high doses, curcumin is poorly absorbed from the gastrointestinal tract, with peak blood levels rapidly reached within one hour after dosing [45]. In rats, after oral administration, plasma curcumin is mainly found in the form of glucuronide conjugates, while just a small amount of the unmodified form is detected. In humans, after curcumin oral administration, glucuronide conjugates and sulfate conjugates are detected in blood, while intact curcumin is barely detected [46].

Ingested curcumin first passes through the stomach, where the absorption of polyphenols is practically absent. Due to its resistance to low pH, curcumin reaches the large intestine without any chemical or structural modifications. In the large intestine, curcumin may be modified by phase I enzymes, a class of enzymes that introduces reactive and polar groups into their substrates. Phase I reactions often produce active metabolites. One of the most common enzymes of this group is cytochrome P450 that catalyzes a substrate hydroxylation. Unexpectedly, curcumin appears not to be metabolized by cytochrome P450, because no products of demethylation or hydroxylation were detected after incubation of curcumin with rat liver microsomes [47]. The phase I metabolism of curcumin includes the successive reduction of the four double bonds. The enzymes responsible for the metabolic reduction have been found to reside in the cytosol of the enterocytes and include the alcohol dehydrogenases [48]. It has been demonstrated that phase I metabolism yielded three metabolites, namely, tetrahydrocurcumin (M1), hexahydrocurcumin (M2), and octahydrocurcumin (M3). Then, curcumin and the phase I metabolites were subject to conjugation via phase II metabolism to yield their corresponding glucuronide and sulfate O-conjugated metabolites.

The result of these reactions is the increase of molecular weight and the production of less active metabolites than their substrates. One of the most important enzymes of this group is the glutathione S-transferase. In vitro and in vivo, curcumin and its reductive metabolites appear to be easily conjugated [32]. Among the reported conjugates, monoglucuronides, monosulfates, and mixed glucuronide/sulfates are included. Glucuronidation is the predominating pathway of conjugation, and the glucuronide of curcumin is usually found as the major metabolite of curcumin in body fluids, organs, and cells [32].

Commercial curcumin also contains demethoxycurcumin and bisdemethoxycurcumin, and these molecules undergo reductive metabolism very similar to curcumin, with the hexahydro product as the major metabolite and much smaller amounts of the octa-, tetra-, and dihydro products.

## 3. Biotransformation of Curcumin by Gut Microbiota

The transformation of curcumin does not occur only by enzymes produced by the enterocytes or by hepatocytes, but also by enzymes produced by the gut microbiota. Gut microbiota can be described as a biological reactor because of its own formidable metabolic functions, like the transformation of numerous compounds that reach the colon. This activity is made possible through the capacity of microorganisms for producing a huge and varied range of enzymes. In particular, curcumin intestinal transformations include several steps and different classes of microbial enzymes.

Thus, the composition of the microbiota will cause different biotransformation of dietary curcumin.

Accordingly, the beneficial effects for consumers depend not only on the polyphenols taken from the diet, but also on the type of microbial population of the individual.

Several enteric bacteria capable of modifying curcumin have been identified.

Curcumin can be modified in the colon tract by a specific microorganism, *Escherichia coli*. The enzyme NADPH-dependent curcumin/dihydrocurcumin reductase (CurA) converts curcumin first into the intermediate, dihhydrocurcumin, and then in the final product, tetrahydrocurcumin, with a two-step reduction that is NADPH-dependent. In the first step of reaction, dihhydrocurcumin is generated from curcumin by reductive destruction of the chromophoric diarylheptatrienone chain. NADPH is an indispensable cofactor for the reduction of curcumin in dihydrocurcumin by CurA. In the second step, dihydrocurcumin is converted in tetrahydrocurcumin with the same mechanism [49].

CurA showed sequence similarity with some enzymes of the medium-chain dehydrogenase/reductase superfamily, which contains many different families including the alcohol dehydrogenases family, implicated in curcumin reduction.

Again, the firmicute *Blautia* sp. (MRG-PMF1), another human intestinal bacteria strain, is involved in curcumin metabolism. This bacterium produces two derivatives, demethylcurcumin and bisdemethylcurcumin by demethylation reaction [50]. Other bacteria capable of modifying curcumin have been identified. *Escherichia fergusonii* (ATCC 35469) and two *E. coli* strains (ATCC 8739 and DH10B) produce dihydrocurcumin, tetrahydrocurcumin, and ferulic acid [51]. Other microorganisms such as *Bifidobacteria longum* BB536, *Bifidobacteria pseudocatenulaum* G4, *Escherichia coli* K-12, *Enterococcus faecalis* JCM 5803, *Lactobacillus acidophilus*, and *Lactobacillus casei* are all biologically relevant bacterial strains capable of degrading curcumin [52].

It has been reported that microbial metabolism of curcumin by *Pichia anomala* or by a bacterial strain of *Bacillus megaterium* DCMB-002 yielded new metabolites through different metabolic processes including hydroxylation, demethylation, reduction, and demethoxylation.

Recently, new curcumin metabolites produced by fecal bacteria have been identified. An analysis performed by using ultra-performance liquid chromatography coupled with quadrupole time of flight mass identified a total of 23 metabolites and discovered different novel human gut microbiota curcumin metabolic pathways, by demethylation, reduction, hydroxylation, and acetylation, or the combination of these [53].

Finally, bacteria from colon may deconjugate glucuronide and sulfate O-conjugated inactive metabolites produced by phase II enzymes and reconvert them to the corresponding phase I active metabolites [48].

Interestingly, there is evidence that curcumin metabolites display a similar or superior potency to curcumin [35]. Indeed, tetrahydrocurcumin possesses superiority over curcumin as a free-radical quencher and it was shown to have therapeutic effects in neurodegenerative diseases. These effects could be due, at least in part, to the inhibition of prominent cytokines’ release, including interleukin-6 (IL-6) and tumor necrosis factor-α (TNF-α), or by inhibition of NF-κB activation.

## 4. Curcumin Effects on Gut Microbiota

Human mucosal surfaces are connected with a diverse microbial community composed primarily by bacteria, but also by viruses, fungi, archaea, and protozoa [54]. The gastrointestinal tract is inhabited by a complex and abundant microbial community, with 100 trillion bacteria, about 10–100 times more than the quantity of eukaryotic cells [55].

**Figure 2:**
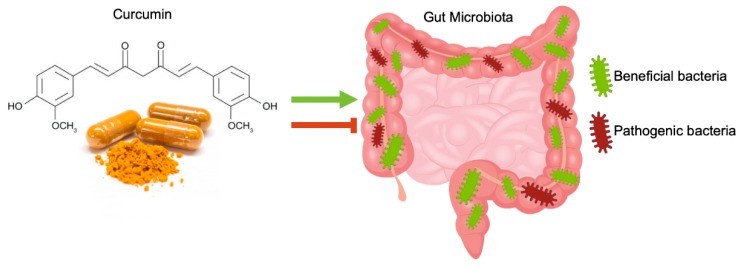
Schematic illustration of the interactions between curcumin and gut microbiota. The administration of curcumin considerably changed the ratio between beneficial and pathogenic bacteria in a gut microbiota community, favoring the growth of beneficial bacteria and limiting the growth of pathogenic ones.

In the intestine, curcumin, after oral or intraperitoneal administration, can exert a regulative effect on the gut microbiota community, affecting microbial richness, diversity, and composition., This effect should be involved in curcumin pharmacological activity [56] (Figure 2).

The administration of curcumin considerably changed the ratio between beneficial and pathogenic bacteria by increasing the abundance of *Bifidobacteria*, *Lactobacilli*, and reducing the loads of *Prevotellaceae*, *Coriobacterales*, *Enterobacteria*, and *Enterococci* [57].

Oral administration of curcumin tends to decrease the microbial richness and diversity in mice [58] and reduce the abundance of several representative bacterial families in gut microbial communities, such as *Prevotellaceae, Bacteroidaceae, and Rikenellaceae,* often associated to the onset of systemic diseases [56,59,60].

This study reported that curcumin administration induced significant weight loss in ovariectomized rats, increasing the number of species of seven different bacterial genus: *Serratia*, *Anaerotruncus*, *Shewanella*, *Pseudomonas*, *Papillibacter*, *Exiguobacterium*, and *Helicobacter*. This study suggests that curcumin can partially reverse changes in the composition of gut microbiota in rats caused by estrogen deficiency induced by ovariectomy [61].

A new pilot work evaluating the effects of curcumin on gut microbiota showed that in the placebo group, there was an overall reduction of bacterial species up to 15%. On the contrary, curcumin-treated subjects showed an increase of bacterial species up of 69%. Subjects responsive to the treatment had uniform increases in *Clostridium* spp., *Bacteroides* spp., *Citrobacter* spp., *Cronobacter* spp., *Enterobacter* spp., *Enterococcus* spp., *Klebsiella* spp., *Parabacteroides* spp., and *Pseudomonas* spp., as well as reduced relative abundance in several *Blautia* spp. and most *Ruminococcus* spp. [62].

Thus, curcumin can change the composition of the microbiota, regulating not only the bacterial populations of the gut, but also the ability of intestinal bacterial strains to produce more active compounds from curcumin itself.

## 5. The Direct Neuroprotective Role of Curcumin and Curcumin Metabolites Produced by Enteric Bacteria

Among the different biological effects exerted by curcumin, the antioxidant one is considered the most interesting in terms of prevention and treatment of neurodegenerative diseases.

Neurodegenerative diseases are characterized by progressive loss of function of a specific population of neurons that determines neural deficit and cognitive impairment. The mechanisms responsible of neurodegeneration have not yet been completely elucidated; however oxidative stress and inflammation are considered the main effectors [63,64,65].

Oxidative stress is generated in the cell when there is imbalance between the amount of reactive oxygen species (ROS) produced and the cellular defense mechanisms that neutralize them.

High levels of ROS irreversibly damage all the cells, but neuronal cells are particularly sensitive also to relatively low levels of ROS. ROS, in fact, are the main factor determining brain ageing and they are involved in the onset and progression of neurodegenerative diseases [66,67].

Curcumin exerts a neuroprotective role by directly or indirectly scavenging free radical species. In fact, curcumin increases significantly the Superoxide dismutase (SOD) activity [68]. SOD, one of major antioxidant enzymes, is able to dismutate superoxide into hydrogen peroxide and oxygen.

Curcumin also shows indirect antioxidant action by elevating catalase plasma activity [69]. Catalases, a class of enzymes able to catalyze decomposition of hydrogen peroxide to water and molecular oxygen, belong to the antioxidant defense system of the cell and protect the cell from oxidative damage by reactive oxygen species.

Different studies indicated that curcumin favors brain health by modulating specific pathways such as: the PI3K/Akt (Phosphatidylinositol 3-kinase/Protein Kinase B) pathway, AMP (AMP-activated protein) kinase pathway, MAPK (Mitogen-Activated Protein Kinase)/Akt pathways [70,71,72] and Akt/Nrf2 (Nuclear factor-E2-related factor 2) pathway [70].

AMPK regulates energy metabolism and plays an important role in the maintenance of cellular homeostasis. This pathway, once activated, protects neuronal damage by decreasing ROS species—ER-associated (Endoplasmic Reticulum) [73].

In neurons, MAPK/Akt and PI3K/Akt represent the major oxidative stress-sensitive signal transduction pathways that regulate cell growth and cell death [74].

Finally, Nrf2 is a key regulator of antioxidant defense during oxidative stress. Activation of Nrf2 upregulates in the neurons defensive antioxidant mechanisms through upregulation of the genes involved in ROS metabolism [75]. When phosphorylated, Akt facilitates Nrf2 nuclear translocation, positively promoting its function [70].

Nitric oxide (NO) is a signaling molecule that shows a key role in inflammation. In physiological conditions, NO has an anti-inflammatory effect. Several studies in humans indicated that curcumin supplementation improves vascular endothelial function in healthy people by increasing NO bioavailability and reducing oxidative stress [41,76].

Thus, curcumin neuroprotective activity seems to be related to the ability of the natural compound to inhibit, directly or indirectly, OS in neurons.

Again, tetrahydrocurcumin is the most studied bacterium-modified curcumin derivative in neuroprotection. It is known that tetrahydrocurcumin reduces oxidative stress and the number of apoptotic neurons, activates autophagy, and inhibits the mitochondrial apoptotic pathway after traumatic brain injury [77]. This metabolite was reported to be protective in vitro against Aβ-oligomer-induced toxicity [78], to modulate in vivo the neuroinflammation, to reduce the level of ROS triggered by β-amyloid fibers, to decrease the mitochondrial membrane potential, and to inhibit caspase activation [79]. In brain injury, tetrahydrocurcumin avoids neuronal cell apoptosis and improves neurobehavioral function by upregulating the Nrf2 pathway [39].

Tetrahydrocurcumin treatment has been reported to be effective also in Parkinson’s disease (PD). A study carried out in a mouse model of PD showed that tetrahydrocurcumin was able to increase dopamine levels and to inhibit the activity of monoamine oxidase, the enzyme that determines the increase of neuronal OS levels through the degradation of neurotransmitters [80,81]. While these results clearly indicate that tetrahydrocurcumin may prevent neurodegeneration [78,82], little is known about the neuroprotective efficacy of others curcumin by-products, such as bisdemethoxycurcumin and demethoxycurcumin and octahydrocurcumin.

Demethoxycurcumin has been reported to exert neuroprotective effects in neuronal cells by glutathione increase and ROS decrease [83,84].

Octahydrocurcumin also represents a very promising antioxidant molecule able to enhance the expression of antioxidant protein through Nrf2 pathway activation [85].

Bisdemethoxycurcumin was reported to show the highest affinity for Aβ-containing plaques in cortical Alzheimer’s Disease brain tissue in comparison with other curcuminoids [86]. Indeed, several in vitro and in vivo experiments suggest that curcumin can also operate by preventing the formation of extra- or intracellular aggregates present in many neurodegenerative disorders.

For prevention and treatment of Alzheimer’s disease, curcumin has been shown to effectively maintain the normal structure and function of cerebral vessels, mitochondria, and synapses, thus reducing risk factors for a variety of chronic diseases and decreasing the risk of Alzheimer’s disease onset [43].

The effect of curcumin on Alzheimer’s disease is related to the modulation of multiple signaling pathways, to its anti-amyloid and metal iron-chelating properties, antioxidation, and its anti-inflammatory activities [87,88].

However, further studies are needed to identify other curcumin metabolites therapeutically active in neuroprotection.

## 6. Curcumin and Gut Microbiota

As detailed before, the paradox of poor bioavailability of curcumin and the wide range of health effects of curcumin can be explained by considering the reciprocal influence existing between curcumin and gut microbiota. Curcumin in the gut favors the growth of beneficial bacteria strains such as Bifidobacteria and Lactobacilli, with reduction of pathogenic strains [56,89]. In addition, curcumin treatment has been found to decrease the microbial richness and diversity, with a specific reduction of species found as cancer-related [90].

Several studies reported that curcumin actively reduces intestinal inflammation by modulating different molecular pathways. Thus, it is possible that curcumin, by modulating the homeostasis of the gut–brain axis, could also determine neuroprotective beneficial [91].

Indeed, dysbiosis is able to induce neuroinflammation, leading to an increased amyloidogenesis or to Lewy bodies accumulation, and to augment specific micro-RNA, down-regulating the triggering receptor expressed in microglial/myeloid cells-2 (TREM2)-mediated amyloid phagocytosis, to reduce, in case of a decrease in butyrate-producing bacteria within the microbiome, the availability of butyrate, an important metabolite known to promote neuron survival [92,93,94].

In particular, gut dysbiosis has been postulated to trigger the onset of some neurodegenerative diseases [95]. Several reports suggest that compromised gut microbiota dysbiosis may represent an important co-factor in Alzheimer’s disease (AD) [96,97]. In fact, different bacterial species are able to produce or aggravate the production of Aβ plaques. Gut dysbiosis, priming the innate immune system by microbiota, determines a neuroinflammatory response that causes misfolding of neuronal amyloid-β and α-synuclein [98].

Gastrointestinal dysfunctions, can be considered as early biomarkers in PD since they are consistently associated with the disease and may precede the classical motor manifestations by decades [99]. In addition, these dysfunctions make the etiology of PD more complicated. It is known that in PD patients, the impairment of the epithelial barrier has been associated with a decrease of Prevotellaceae. These strains are the main producers of mucin, a protein that protects the epithelium from pathogens [100].

More interestingly, in PD patients, there is a decrease of butyrate, a histone deacetylase inhibitor which protects dopaminergic neurons from degeneration by upregulating the neurotrophic factors, such as BDNF (Brain Derived Neurotrophic Factor) and GDNF (Glial Cell Line-derived Neurotrophic Factor) [101].

Studies in humans and in Huntington Disease (HD) animal models also reveal gastrointestinal dysfunctions, which may contribute to the worsening of the disease. The consistent loss of body weight—the most important non-neurological complication of HD and a direct consequence of gastrointestinal defects—has been associated with an impaired gut mobility and malabsorption [102]. Gut microbiota could have a role in HD since presymptomatic HD subjects display serum metabolomic shifts that suggest changes in gut microbial-derived metabolites [103].

Efforts to modulate gut microbiome in cases of neurodegeneration are limited and involve the use of antibiotics, fecal microbiota transplant, prebiotic/probiotic supplementation, and dietary interventions. In this context, curcumin can represent a potential therapeutic option against neurodegeneration since it exerts beneficial effects on gut microbiome, without any apparent toxicity, restoring the dysbiosis within patients suffering from neurodegenerative diseases and maintaining a proper microbiota–gut–brain axis.

These suggestions may be promising to unravel new therapeutic strategies for neurodegenerative diseases. It is known that neurodegeneration determines an imbalance in gut microbiota metabolism that results in changes of endocrine signaling in the host [104]. Further analysis on the microbiota composition and administration of specific curcumin neuroactive metabolites will be helpful in the identification of novel targeted treatments active in neuroprotection.

## 7. Conclusions

Curcumin represents one the most studied herbal remedies. It is generally considered the main component of turmeric responsible for the different pharmacological activities. However, curcumin is characterized by low systemic bioavailability and rapid metabolism. To address its pharmacological and therapeutic advantages, it is fundamental to consider curcumin interplay with gut microbiota that might pave the way to fill the gap between the low bioavailability and the wide health effects.

In fact, gut microbiota impact on curcumin metabolism, providing active metabolites. On the other hand, curcumin can influence gut microbiota composition, allowing the growth of strains needed to maintain correct host physiologic functions. This is the case of neurodegenerative diseases in which often a gut dysbiosis precedes the onset of the clinical signs.

Curcumin metabolism can be different among individuals, since everyone has his/her own microbiota compositions. Thus, the beneficial effects can be different due to the individual different bacterial content. Analysis of gut microbiota changes in health and diseases in the presence of curcumin will allow to identify bacterial strains in curcumin conversion.

The results summarized in the review suggest that curcumin alone can exert a neuroprotective function by affecting different neuropathological pathways. The role of microbiota in enhancing these positive effects could be related both to the production of metabolites more active and with better pharmacokinetics and to the modification of microbiota composition, with a prevalence of the healthy gut bacteria, like *Bifidobacteria* and *Lactobacilli*.

Additional studies, especially in humans will be necessary to unravel in depth the modification of microbiota composition achieved by curcumin. This will lead to an understanding of strategies needed for delivering health benefits by microbiota modulation and represents the first step to considering novel therapeutic applications of curcumin, gut microbiota-based.

Modification of microbiota and its metabolites will provide a new consideration for novel therapeutic intervention in neurodegenerative diseases.
